# Efficient Plant Production of Recombinant NS1 Protein for Diagnosis of Dengue

**DOI:** 10.3389/fpls.2020.581100

**Published:** 2020-10-26

**Authors:** Mariana Fonseca Xisto, Roberto Sousa Dias, Elias Feitosa-Araujo, John Willians Oliveira Prates, Cynthia Canedo da Silva, Sérgio Oliveira de Paula

**Affiliations:** ^1^Department of General Biology, Federal University of Viçosa, Viçosa, Brazil; ^2^Department of Plant Biology, Federal University of Viçosa, Viçosa, Brazil; ^3^Department of Microbiology, Federal University of Viçosa, Viçosa, Brazil

**Keywords:** dengue, NS1 protein, *Arabidopsis thaliana*, diagnosis 2, plant expression

## Abstract

Dengue fever is endemic in more than 120 countries, which account for 3.9 billion people at risk of infection worldwide. The absence of a vaccine with effective protection against the four serotypes of this virus makes differential molecular diagnosis the key step for the correct treatment of the disease. Rapid and efficient diagnosis prevents progression to a more severe stage of this disease. Currently, the limiting factor in the manufacture of dengue (DENV) diagnostic kits is the lack of large-scale production of the non-structural 1 (NS1) protein (antigen) to be used in the capture of antibodies from the blood serum of infected patients. In this work, we use plant biotechnology and genetic engineering as tools for the study of protein production for research and commercial purposes. Gene transfer, integration and expression in plants is a valid strategy for obtaining large-scale and low-cost heterologous protein production. The authors produced NS1 protein of the dengue virus serotype 2 (NS1DENV2) in the *Arabidopsis thaliana* plant. Transgenic plants obtained by genetic transformation expressed the recombinant protein that was purified and characterized for diagnostic use. The yield was 203 μg of the recombinant protein per gram of fresh leaf. By *in situ* immunolocalization, transgenic protein was observed within the plant tissue, located in aggregates bodies. These antigens showed high sensitivity and specificity to both IgM (84.29% and 91.43%, respectively) and IgG (83.08% and 87.69%, respectively). The study goes a step further to validate the use of plants as a strategy for obtaining large-scale and efficient protein production to be used in dengue virus diagnostic tests.

## Key Message

This study goes a step further to validate the use of plants as a strategy for obtaining large-scale and efficient protein production to be used in dengue virus diagnostic tests.

## Introduction

Dengue virus (DENV) infection is one of the most important human diseases transmitted by arthropods. Virus transmission is performed by mosquito vector, with predominance of the genus *Aedes* (Bhatt et al., 2013). Like other emerging infectious diseases, outbreaks of the DENV are unpredictable and potentially widespread, and can result in epidemics that threaten global public health (Fauci and Morens, 2012; Morens and Fauci, 2013; McArthur, 2019). The disease is endemic in over 120 countries, which account for 3.9 billion people at risk of infection worldwide (World Health Organization, 2020). From 1998 to 2018, more than 20 million cases were recorded in the Americas, 65% of which were reported in Brazil (Pan American Health Organization, 2019).

The DENV belongs to the *Flaviviridae* family and has four antigenically distinct serotypes, DENV1, DENV2, DENV3, and DENV4 (Lindenbach and Rice, 2003; Best, 2016). The DENV viruses are enveloped, with a single-stranded RNA genome with positive sense. Genome translation results in three structural proteins (envelope protein – E, membrane protein – M, and capsid protein – C) and seven non-structural proteins (NS1, NS2a, NS2b, NS3, NS4a, NS4b, and NS5). Non-structural (NS) proteins are related to viral replication, protein expression and virulence of serotypes (Heinz and Stiasny, 2012; Simmonds et al., 2017).

Non-structural 1 is a highly conserved glycoprotein among all *Flaviviruses*, with molecular weights ranging from 46 to 55 kDa, depending on the glycan pattern (Winkler et al., 1988; Pryor and Wright, 1994). Correct glycosylation is related to its efficient secretion, virulence, and virus replication. Protein can be found in three forms: monomer, dimer (membrane anchored), and hexamer (secreted) (Noisakran et al., 2008; Chuang et al., 2013; Muller and Young, 2013). The different actions attributed to NS1 include the activation of toll-like receptors (TLRs), complement system inhibition, and immune response induction linked to the acute phase in severe cases (Libraty et al., 2002; Avirutnan et al., 2010; Rastogi et al., 2016; Modhiran et al., 2017): it is the first viral protein present in the bloodstream of infected patients, and is currently used as a biomarker for early diagnosis in the acute phase (Shu et al., 2009).

The first licensed tetravalent vaccine, Dengvaxia (Sanofi Pasteur), presented complications in protecting individuals who had never had the disease before, resulting in a WHO alert with recommendations for further testing (Guy et al., 2015; Iacobucci, 2018). A problem presented in recent years is the occurrence of dengue infections in regions where cases had not yet been reported. The spreading of the virus is cause for alarm, and intensifies the need to control infections through correct diagnosis, avoiding underreported cases. Since many infected patients are asymptomatic and present non-specific conditions, it is not possible to rely solely on clinical manifestations, which reinforces the need for differential molecular diagnosis (Mardekian and Roberts, 2015; Katzelnick et al., 2017; Muller et al., 2017; Wiemer et al., 2017). Therefore, a quick, accurate and low-cost diagnosis is essential to confirm suspicions of dengue cases, favoring the adequate treatment of infected patients, especially in countries with limited health care resources.

Due to the lack of a vaccine with effective protection against all the four serotypes of the virus (Shrivastava et al., 2017), differential molecular diagnosis is the best and safest alternative for the correct treatment of the disease (Pang et al., 2017). As recommended by the WHO Special Program for Research and Training in Tropical Diseases (TDR), the specifications of an ideal test for dengue should address the following points: (i) high sensitivity and specificity in detection, (ii) fast results, and (iii) low cost (Peeling et al., 2010).

Detection of anti-dengue antibodies in serum samples is widely used, especially in developing countries, due to its ease of use compared to other techniques such as viral RNA detection. In the primary infection with DENV, IgM response has higher titers and is more specific than during subsequent infections. In contrast, the IgG titer is higher in a second infection. Several IgM and IgG capture ELISA kits are commercially available, with sensitivity ranging from 21 to 99% and 8 to 89%, respectively, and specificities varying from 52 to 100% for IgM and 63 to 100% for IgG. The antigens used in the commercial kits are obtained from virus culture in mouse brain, Vero cells and C6/36 mosquito cells (Groen et al., 2000; Pal et al., 2014; Fatima and Wang, 2015; Guerrero and Bello, 2019), which require high levels of biosafety and make them expensive to prepare. In non-endemic regions, IgM-based tests can be used in clinical surveillance, with a high probability that positive results indicate recent infections (during the last 2–3 months) (Dutra et al., 2009; Tuan et al., 2015; Wang et al., 2015; Pang et al., 2017).

Plant biotechnology in recent years has evolved significantly with the use of genetic engineering as a tool for protein production, both for research and commercial purposes. Gene transfer, integration and expression in plants is a valid strategy for obtaining large-scale and low-cost heterologous proteins (Schillberg et al., 2019). In addition to transgenic systems, other plant production techniques are being explored, including transient expression systems, inducible expression systems, and protein targeting (Twyman et al., 2003; Schillberg et al., 2013; Spiegel et al., 2015). In transient expression, exogenous DNA is not integrated into the host plant genome. It generates results in a shorter time; however, in stable expression, the hosts can be used for several generations and for a longer time. Furthermore, new transfections are not necessary (Schillberg et al., 2019). Compared with other organisms, plants have advantages because they are eukaryotes with a mammalian-like post-translational processing system. Therefore, proteins that need modification, such as glycosylation, are more accurately represented in these organisms (Obembe et al., 2011; Kim et al., 2014; Ko, 2014).

The production of viral proteins in plants is commercially attractive because of the facility for increasing the scale of production and purification, with plants functioning as bioreactors. In addition to presenting advantages in terms of biosafety, plant production has a low cost (Commandeur et al., 2003). Since the first protein expressed in tobacco by Barta et al. (1986), research has evolved, and a large number of products with therapeutic importance, such as vaccines, antibodies and proteins with different purposes, have been expressed in plants, using different strategies and plant hosts (Mason et al., 1992; Mett et al., 2008; Demain and Vaishnav, 2009; Bock and Warzecha, 2010; Gomord et al., 2010; Vojta et al., 2015).

In this work, *Arabidopsis thaliana* was chosen because it is a small herbaceous plant, with short generation time and large seed production. The genome has a relatively small size and ease of transformation by *Agrobacterium tumefaciens* (Hwang et al., 2017). As a further advantage, the transformed plant functions as a bioreactor and can be cultivated in confined spaces, such as a controlled growth chamber or greenhouse (Lloyd et al., 1986; Feldmann and Marks, 1987; Chang et al., 1994; Lacroix and Citovsky, 2013; Pitzschke, 2013; Von Schaewen et al., 2018).

After genetic transformation, a clone with the DENV2 non-structural protein 1 (NS1) gene introduced into the genome was selected. From this, we purified and characterized the recombinant protein, which presented antigenic potential, with the ability to recognize anti-dengue antibodies in the serum of infected patients with high specificity and sensitivity in enzyme-linked immunosorbent assays (ELISA). The study takes a step forward by using the plant as the key part of a valid strategy for large-scale and efficient production of a protein that will be used in serological tests, since the production of this antigen is still a key point that generates high cost in commercial diagnosis kits.

## Materials and Methods

### NS1DENV2 Expression Cassette Construction

**FIGURE 1:**
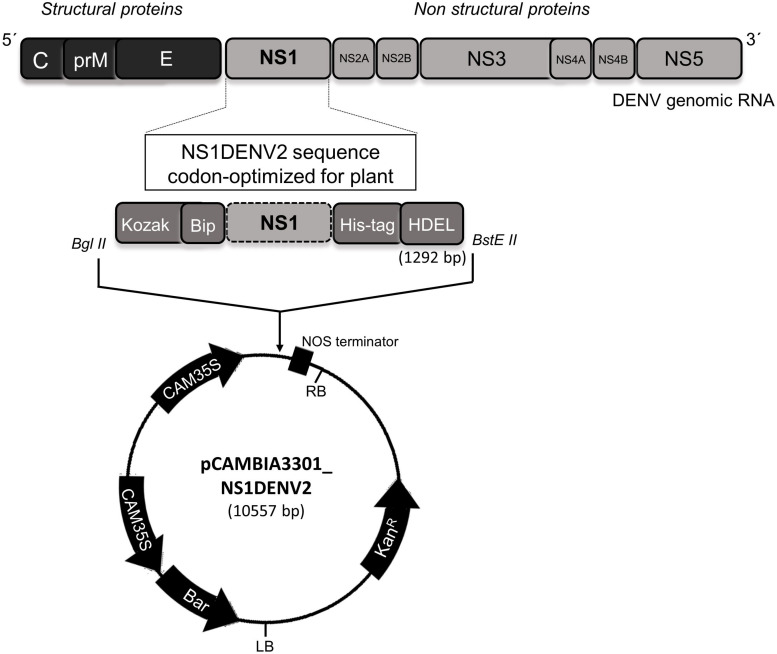
Construction of the pPCAMBIA3301_NS1DENV2 vector.

Non-structural protein 1 (NS1) from DENV2 strain New Guinea C (GenBank access: MK506264) was codon-optimized for expression in *A. thaliana* and synthesized by GenScript^TM^. The cassette inserted into the pUC57 vector was constructed with: (i) the restriction sites for *Bgl II* and *BstE II* enzymes flanking the gene (for cloning); (ii) Kozak sequence (ribosome recognition site in plant eukaryotic cells) (Kozak, 1978, 2002; Nakagawa et al., 2008); (iii) signal peptide Bip At5g44620 (targeting to the rough endoplasmic reticulum – RER) (Lee et al., 2011); (iv) polyhistidine tag (to purification step) (Hochuli et al., 1987; Terpe, 2003); and (v) HDEL sequence (RER membrane attachment) (Munro and Pelham, 1987; Napier et al., 1992; Koizumi, 1996), totaling 1292 bp. Restriction enzymes (Bgl II and BstE II) were used to cut and clone the insert into the expression vector pCAMBIA3301 (Figure 1).

### Bacterial Strains and Vectors

The cloning vector obtained, pUC57_NS1DENV2, was maintained in *Escherichia coli* TOP10F. Binary vector pCAMBIA3301 has the herbicide resistance gene (bar) to phosphinothricin (PPT) and the kanamycin resistance gene as the selection markers. Subsequent to the cloning of the NS1DENV2 gene into the expression vector (pCAMBIA3301_NS1DENV2), the competent *A. tumefaciens* strain GV3101 was transformed by electroporation (McCormac et al., 1998), grown at 28°C on YEB medium (0.5% m/v peptone, 0.1% m/v yeast extract, 0.5% m/v meat extract, 0.5% m/v sucrose, and 0.024% m/v MgSO_4_, pH 6.8) with gentamycin (100 μg/ml) and kanamycin (50 μg/ml) antibiotics, and positive colonies were selected.

### Detection and Cloning Confirmation

In the polymerase chain reaction (PCR), a pair of primers, pCambNS1s (5′-GGAGATCTATGGATAGTGGTT GCGTTGTGA-3′) and pCambNS1as (5′-GGGGTAACCTGAG GCTGTGACCAAGGAGT-3′), was used to confirm positive colonies, flanking the NS1DENV2 gene region, resulting in a 1056 bp fragment. The GoTaq^®^ DNA Polymerase kit (Promega^TM^, United States) was used, with an initial step of 94°C for 5 min, 35 cycles of 95°C for 1 min, 55°C for 2 min and 72°C for 2 min, and a final step of 72°C for 10 min.

The digestion reaction was performed using restriction enzymes *Bgl II* and *BstE II* (Thermo Fisher^TM^, United States), which flank the NS1DENV2 gene and generate a fragment with 1292 bp. The double digestion reaction was incubated at 37°C for 12 h. For cloning, the linearized expression vector and the insert were ligated using the T4 DNA Ligase kit (Promega^TM^), purified from 1% agarose gel using the QIAquick Gel Extraction kit (QIAGEN^TM^, Germany).

### *Arabidopsis thaliana* Transformation Using *A. tumefaciens*

The wild type (WT) *A. thaliana* Columbia (Col-0) seeds were incubated using a photoperiod with 16 h exposure to light (200 μmol/m^2^s) and 8 h dark at 25°C on a composite substrate (organic substrate and thick vermiculite, 2:1). After germination and vegetative growth until the emergence of the first flowers, the main shoot was removed to produce the emergence of a larger number of lateral shoots. Plants with lateral shoots with approximately 45 days in the early stage of flowering were used for transformation. The *A. tumefaciens* culture (grown at 28°C, 150 rpm, OD = 0.4/0.5, at 600 nm absorbance) with vector pCAMBIA3301_NS1DENV2 was used to perform floral-dip (Clough and Bent, 1998), a method that involves immersing the flower buds in the bacterial suspension three times in a 7-day cycle. Plants were kept in a dark environment for 24 h, after which they were subjected to the ideal conditions for vegetative growth until the reproductive cycle was complete for maturation and seed collection.

### Clone Selection

All generations were obtained for self-fertilization. T1 seeds (first generation, collected from plant T0, the initial transformed generation) were sterilized (sodium hypochlorite and autoclaved distilled water, 1:1) and seeded with MS medium (Murashige and Skoog, 1962) with phosphinothricin (7.5 μg/mL) for selection of transgenic plants. They were then incubated at 4°C in the dark for 72 h to break dormancy, and then placed in the growth chamber with a photoperiod of 8 h of light and 16 h of darkness at 24–26°C for germination. After 10 days, the seedlings that emerged were selected and transplanted into composite substrate until the next generation of seeds appeared. The T2 seeds were cultured as before until the T3 generation, and the dominant homozygous plants for the NS1DENV2 transgene were selected, cultured and stored at −80°C for further analysis.

At 40 days of age, the leaves of the T3 plants were macerated in nitrogen and the genomic DNA of the clones was extracted by the method described by Edwards et al. (1991) in triplicate. PCR was done to confirm the insertion of the transgene using the same primers previously employed for cloning confirmation. One of the clones was selected for the following steps.

### Total Protein Extraction

For each 1 g of fresh leaf (both transformed and WT, which served as control) with 40 days of age, macerated in liquid nitrogen, 5 mL of extraction buffer (100 mM Tris–HCl, 1 mM EDTA, 2% SDS, pH 8.0) was added, containing 0.1 mM PMSF (protease inhibitor) and 0.2 g PVPP (Polyvinylpolypyrrolidone). Then, leaf extracts were sonicated on ice for 10 s at 30% amplitude, centrifuged at 12,000 × *g*, and the supernatant was stored at −80°C.

### Protein Purification

After total protein extraction, the supernatant was diluted in binding buffer (sodium phosphate 20 mM, NaCl 500 mM, imidazole 20 mM, pH 7.4) and purified by affinity chromatography. For each 1 ml of extraction supernatant, 1 ml of binding buffer was added. A 5 mL HisTrap^®^ Fast Flow Crude column (GE HealthCare^TM^, Sweden), previously equilibrated with the binding buffer, was coupled to the AKTA^®^ purification system (GE Healthcare^TM^, Sweden). The flow rate was 0.5 mL/min and the retention time in the column was 10 min/mL. After injection of total protein into the system and its flow through the column to bind it, it was washed with a 10X sample volume with binding buffer. The column-bound protein fraction was recovered using a pH 7.4 elution buffer (20 mM sodium phosphate, 500 mM NaCl, 400 mM imidazole). After purification the sample was filtered using Amicon^®^ Ultra-15 Centrifugal Filter Units 10,000 NMWL to remove imidazole, using sodium phosphate buffer (20 mM). Then it was lyophilized and solubilized in 1 mL of Tris–HCl buffer (100 mM), pH 8.8. Total quantification was performed using the BCA kit (Pierce Chemical Co., United States). We estimated the specific concentration of NS1DENV2 by densitometry of bands in the gel with a standard curve of BSA. For this, we use the ImageJ software.

### Western Blot

The protein extract (10 μL) was submitted to electrophoresis in acrylamide gel (SDS-PAGE 12%) and stained with Coomassie G-250. To perform the immunoassay the NS1-DENV2 protein was transferred to a nitrocellulose membrane. As a primary antibody, a polyclonal pool from three patients positive to the dengue disease was used. A goat-produced polyvalent anti-human IgM conjugated with peroxidase was used as a secondary antibody (Sigma-Aldrich^TM^, Brazil).

The dot-blot was made from one drop (5 μL) of the crude extract of the transformed plant (P3) and the WT plant on a nitrocellulose membrane, using the same steps as western blot.

### Immunolocalization

Confocal laser scanning microscopy (CLSM) with the LSM 510 (Zeiss^®^, Germany) at the Microscopy and Microanalysis Nucleus (UFV) was used for the immunolocalization assay. One leaf of each plant, at 30 days of age, was used for *in situ* NS1DENV2 protein detection (Sauer et al., 2006).

After being fixed in 4% paraformaldehyde, both transgenic and wild plant tissues were labeled with primary anti-His tag monoclonal antibodies (Sigma-Aldrich^TM^, Brazil) (1:500, v/v) in 1X PBS (0.02% KCl, 0.8% NaCl, 0.18% Na_2_HPO_4_._2_H_2_O, 0.024% KH_2_PO_4_, pH 7.4), and sequentially, tissues were incubated with FITC-conjugated secondary antibody (Sigma-Aldrich^TM^, Brazil) (1:500: v/v) in 1X PBS solution, for 2 h at 37°C. Then, To-Pro3 nucleic acid dye (Thermo Fisher^TM^) was added and the slides were assembled in Mowiol (Sigma-Aldrich^TM^, Brazil), analyzed and photographed.

### Serum Samples

For immunoassays, the serum from 253 patients from the *Laboratório Central de Saúde Pública do Estado de Rondônia* (Rondônia State Laboratory for Attention to Public Health, LACEN/RO) and the *Banco Central de Sangue do Estado de Rondônia, Brazil* (Rondônia State Central Blood Bank, FHEMERON/RO) was used. All serum was tested and used according to protocols approved by the Ethical Committee and Institutional Committee of Human/Animal Care and Use.

Samples were previously confirmed as positive for DENV IgM or IgG by IgM MAC-ELISA (Pan-Bio^TM^, Australia) and Capture Duo IgM and IgG ELISA Kit (Sanofi^TM^, EUA).

### ELISA Assay

Recombinant NS1-DENV2 protein was used as an antigen coating to sensitize 96-well high-binding ELISA plates (JETBiofil^TM^, Korea) at a 1 μg/well concentration in a carbonate-bicarbonate buffer, pH 9.6. The patient serum samples were diluted 1/100, added in duplicates in the plates and incubated at 37°C for 60 min. Peroxidase-conjugated IgM (Sigma-Aldrich^TM^, Brazil) and anti-human IgG (Sigma-Aldrich^TM^, Brazil) secondary antibodies were added to the respective plates at 1/2500 dilution and incubated at 37°C for 60 min. After this incubation time, an ABTS substrate (2,2′-Azinobis [3-ethylbenzothiazoline-6-sulfonic acid]-diammonium salt) (Sigma-Aldrich^TM^, Brazil) was added and reaction was blocked with a 2M solution of H_2_SO_4_. The absorbances were read by spectrophotometer (Multiskan GO, Thermo Fisher^TM^) at a wavelength of 450 nm.

### Statistical Analysis

Receiver operating characteristic (ROC) curves were analyzed to estimate diagnostic cutoff, sensitivity and specificity using GraphPad Prism version 7.00 for Windows (GraphPad Software, United States). The mean of ELISA duplicates was used to determine the number of points in the curve (270 points for IgM and 222 points for IgG).

In this study, all experiments were performed with three biological replicates and three technical replicates per biological replicate.

## Results

### Sequence Cloning and *A. tumefaciens* Transformation

Cloning and expression vectors pUC57_NS1DENV2 and pCAMBIA3301_empty were extracted from stored *E. coli* TOP10F to be digested by restriction enzymes *Bgl II* and *BstE II* (Supplementary Figures 1A,B). A NS1DENV2 fragment from pUC57_NS1DENV2 was inserted into pCAMBIA3301, forming pCAMBIA3301_NS1DENV2, which was transformed in *E. coli* TOP10F again and stored. In order to confirm cloning, a double digestion of the two clones – which grew in the presence of antibiotics – was performed (Supplementary Figure 1C).

The binary vector pCAMBIA3301_NS1DENV2 extracted from *E. coli* TOP10F was used to transform *A. tumefaciens*. Five *Agrobacterium* transformants were selected and confirmed by double digestion of the extracted plasmid DNA (Supplementary Figure 1D).

### Transformation and *A. thaliana* Genetic Segregation

After *A. thaliana* WT germination and the appearance of lateral flowering shoots, the first cycle of the “floral-dip” method for plant genetic transformation was carried out. The procedure was repeated two more times, and the T^0^ plants (initial transformants) were kept in the growth chamber until the reproductive cycle was completed for maturation, drying of the siliqua and collection of the T1 seeds.

The first generation (T^1^) of transformed *A. thaliana* seeds was grown on a solid MS medium containing phosphinothricin (PPTR) as a selective agent, and the transformation efficiency was 3.59% (306 seeds plated – 11 seeds germinated).

**FIGURE 2:**
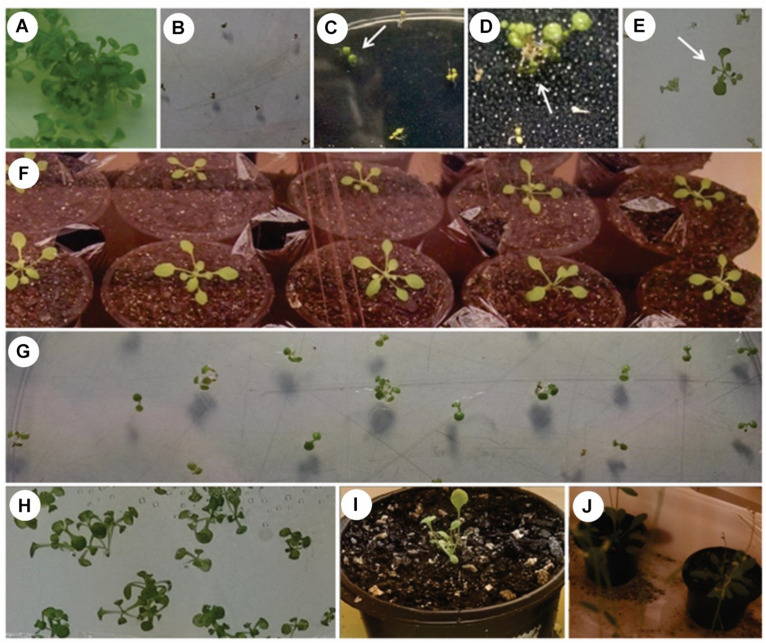
Selection of *Arabidopsis thaliana*. **(A)** WT plant in MS medium without PPT; **(B)** WT plant in MS medium with PPT; **(C–E)** Germination of the *A. thaliana* transformed T^1^ generation (white arrow indicate seedlings with normal vegetative growth); **(F)** Transformed plants of generation T^2^; **(G)** Germination of 100% of the seeds in dominant homozygosis; **(H)** Transformed plants of generation T^3^ seeds; **(I,J)** Cultivation of the transformed plant until the production of seeds (60 days of age).

The T^1^ seeds that germinated into healthy seedlings presented superior vegetative development, visually noticeable from the tenth day, compared to the seeds that could not develop vigorously (Figures 2A–E). Healthy seedlings had a green coloration, and physiological characteristics of the normal vegetative state of the *A. thaliana*. These differed from the other seeds that did not germinate or that gave rise to seedlings which did not develop and presented a yellowish coloration, with altered growth in vegetative state as a consequence of the herbicidal action.

From seeds of the second generation, T^2^, a total of 11 seedlings were generated, which were transplanted to pots with a composite substrate and maintained in a growth chamber (Figure 2F). Of these 11 seedlings, nine reached the complete stage of the reproductive cycle with inflorescence formation, maturation and development of the embryos producing siliqua with seeds, around 60 days of age. Seeds of the second generation (T^2^) (nine plants) were collected and cultured in a solid MS medium with the phosphinothricin selective agent (PPT^*R*^).

From the last generation (T^3^) of cultivated *A. thaliana*, three plants (P1–P3) presented genetic segregation characteristic of dominant homozygosity, with approximately 100% seed growth (Figure 2G). These were grown until the production of seeds (Figures 2H–J). At the end of this period, these seeds were collected and stored at 4°C.

### NS1DENV2 Expression

**FIGURE 3:**
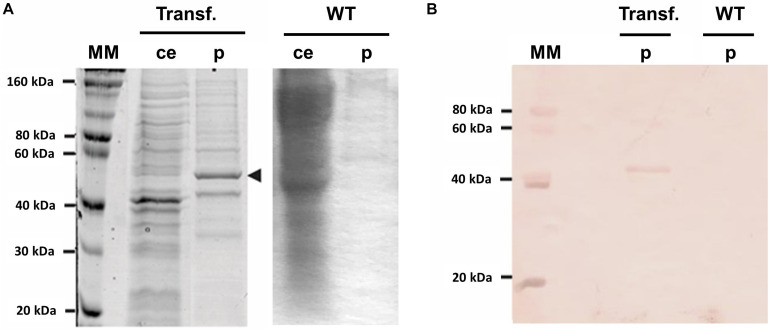
Confirmation of genetic transformation of *Arabidopsis thaliana*. **(A)** Protein gel, SDS-PAGE 12% – (MM) molecular marker protein, (ce) crude extract, (p) purified fraction (black arrow head indicating NS1DENV2 protein) around 50 kDa, (Trans.) transformed and (WT) wild-type; **(B)** Western blot – (MM) molecular marker protein, (p) purified protein fraction, (Trans.) transformed and (WT) wild-type. Protein labeled with anti-dengue positive antibody in the fraction of the transformed plant. All experiments were performed with three biological replicates and three technical replicates per biological replicate.

A transgenic plant (P3) producing the recombinant protein NS1DENV2 was submitted to total proteins extraction and purification by chromatography. SDS-PAGE electrophoresis analysis (Figure 3A) followed by western blotting (Figure 3B) was done using serum samples previously confirmed as positive for DENV infection. The immunolabeled band was around 50 kDa and was differentially expressed compared to *A. thaliana* WT. Quantification of total protein in crude extracts and the purified fraction was performed by BCA method (Table 1). The quantification of the recombinant protein yield was performed using a bovine serum albumin (BSA) to obtain a standard curve. From the curve, the pixel density was obtained for each concentration, using the ImageJ software^1^, and NS1 protein pixel density was interpolated with the standard curve. For each 1 g of fresh leaf used in the protein extraction, 1 mL of total proteins was obtained at the end of the purification process, solubilized in Tris–HCl buffer. Therefore, the yield obtained after purification on a nickel column was estimated using the densitometry approach to provide the 203 mg of NS1DENVV per kilogram of fresh leaf. The purity obtained was 76.58%, indicating the percentage of recombinant protein present in the sample after purification in a chromatographic column (Table 2 and Supplementary Figure 2).

**TABLE 1:** Total protein quantification and purification yield of leaf extracts from transformed and WT plants, using BCA method.

	Transformed		WT	
	Crude extract	Purified	Crude extract	Purified
Abs^562nm^	0.524	0.221	0.497	0.171
[ ] μg/mL	1022.75	265.25	955.25	140.25
Yield (mg/mL)	5113.75	265.25	4776.25	140.25

*Quantification of total protein (BCA kit): Standard curve equation: y = 0.0004x + 0.1149. R^2^ = 0.9963.*

**TABLE 2:** Quantification of NS1DENV2 protein by densitometry.

	Densitometry	[ ] μg/mL	Yield (mg/kg)	Purity (%)
Purified (p)	74279.170	203.1317	203.1317	76.58

*Yield per kilogram of fresh leaf. BSA standard curve: y = 364.05x + 329.06. R^2^ = 0.9863.*

### Comparative Confocal Laser Scanning Microscopy

**FIGURE 4:**
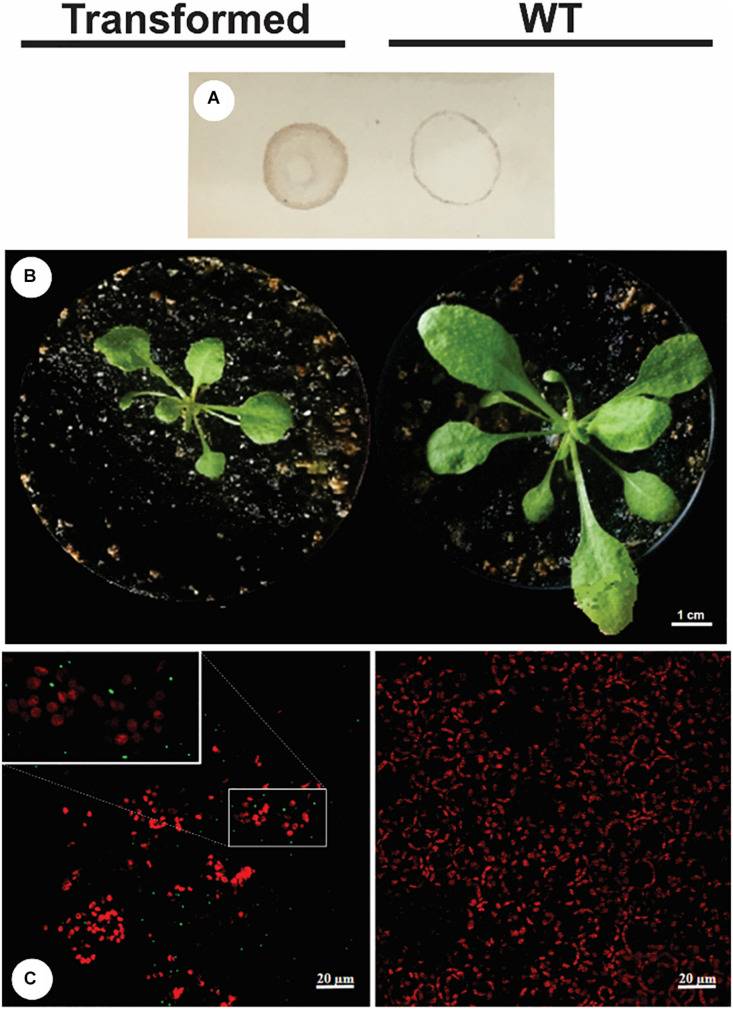
Transformed plant vs. WT plant (28 days of age). **(A)** Dot blot; **(B)** Photo of the rosette in vegetative growth; **(C)** Confocal microscopy – Transformed **(left bottom)** and WT **(right bottom)**. All experiments were performed with three biological replicates and three technical replicates per biological replicate.

**FIGURE 5:**
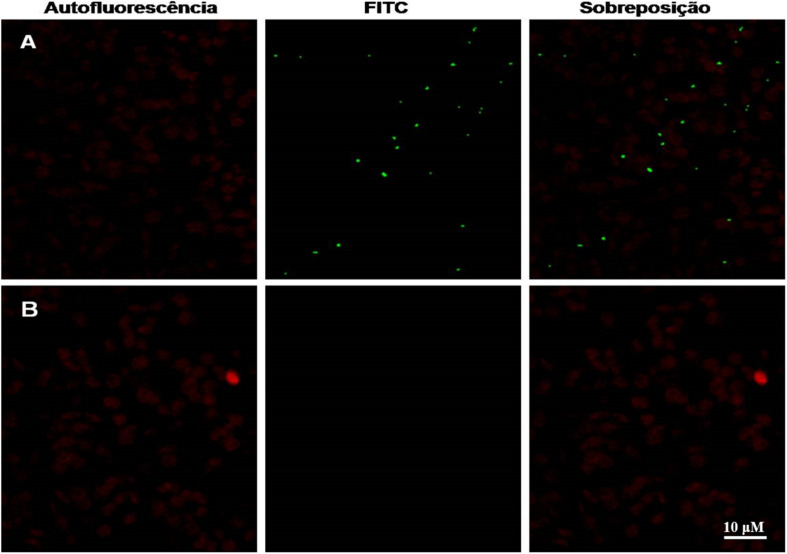
Confocal microscopy. NS1DENV2 recombinant protein (green) spread throughout the leaf, labeled with FITC in the transformed plant, and in red the chlorophyll autofluorescence. All experiments were performed with three biological replicates and three technical replicates per biological replicate. **(A)** Transformed plant; **(B)** WT plant.

After 30 days of growth, a lower vegetative development was observed in transgenic plant P3 in relation to the WT plant, with a smaller rosette diameter and leaf area observed and a comparative dot-blot was made with the crude extract of each plant (Figures 4A,B). NS1DENV2 protein *in situ* immunolocalization in the P3 plant was done by confocal microscopy and compared to the wild plant (WT) (Figure 4C). Green fluorescent points were observed throughout the leaf tissue of the P3 plant in the form of oval protein bodies, which are clear evidence of the presence, expression and localization of NS1DENV2 protein within the plant cells of the transgenic *A. thaliana*. On the right side, in the wild type plant, green fluorescence was not detected, indicating the absence of immunostaining. Red dots observed are produced by the autofluorescence of chlorophyll present in chloroplasts which remained in the tissue after sample preparation (Figure 5).

### Indirect ELISA

**FIGURE 6:**
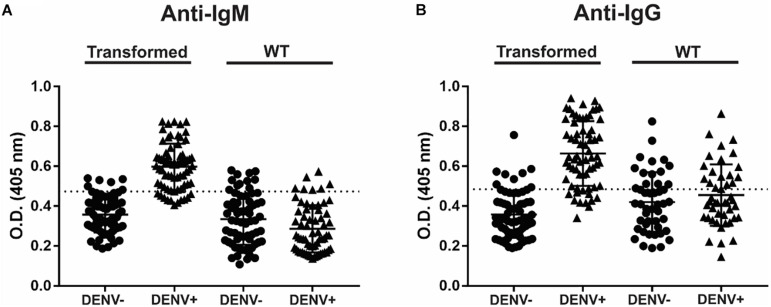
Anti-dengue indirect ELISA, using purified plant protein as antigen to capture. **(A)** Anti-IgM and **(B)** Anti-IgG.

To detect IgM and IgG anti-dengue antibodies, the recombinant NS1DENV2 protein extracted from transgenic *A. thaliana* was used as the antigen in the tests, in exactly the same way as for the test plant. For anti-dengue IgM, 84.29% sensitivity, and 91.43% specificity were obtained with a cut-off value of 0.4737. For anti-dengue IgG, 83.08% sensitivity and 87.69% specificity were obtained with a cut-off of 0.4847 (Table 3). The graphs were reproduced from the ROC curve with 95% CI (confidence interval) and set the best cut-off for each assay (Figure 6). From the 253 anti-dengue serum samples (132 positives and 121 negatives), we demonstrated the numbers of false positives, false negatives, true positives, and true negatives (Table 4) for comparative evaluation. This survey was performed for each assay (IgM and IgG) using the samples tested on the transformed plant and WT plant.

**TABLE 3:** Sensitivity and specificity values of the anti-dengue indirect ELISA (IgM and IgG).

	% Sensitivity	% Specificity
Anti-IgM	84.29	91.43
Anti-IgG	83.08	87.69

*Receiver operating characteristic (ROC) curves were analyzed to estimate the diagnostic sensitivity and specificity.*

**TABLE 4:** Numbers of the true and false positive and negative samples according to the protein extract used (transformed and WT plants).

	Anti-IgM		Anti-IgG	
	Transformed	WT	Transformed	WT
False positive	10	28	15	90
False negative	22	–	22	–
True positive	110	–	110	–
True negative	111	225	106	163

*(n = 253 patients) 132 positives and 121 negatives.*

## Discussion

The results presented in this work are promising as a valid strategy for further studies to obtain protein on a large-scale and in an efficient form that can be used in the dengue serological tests. Confirmation of the NS1 gene cloning of DENV2 (NS1DENV2) in *A. thaliana* generated a functional transgenic clone, indicating that the insertion did not occur in key genes, rendering them inactive. In the first step, the gene was optimized for expression in plants, and was added to specific sequences to obtain higher levels of NS1DENV2 protein expression. Sequences added (Kozak sequence) in the expression cassette of the present work are involved in translation mechanisms and specific localization of proteins within the plant cells. The addition of small signal sequences (signal peptide, Bip and HDEL) in the cassette has the function of ensuring the address and retention of the protein in the rough endoplasmic reticulum, avoiding the protein degradation which would occur if these were free circulating in cytoplasm or an unwanted export to the outside of the cell (Benchabane et al., 2008; Pillay et al., 2014).

Wild type (WT) *A. thaliana* seeds were grown and the plants with the highest number of floral shoots were used for *A. tumefaciens*-mediated gene transformation containing the plasmid pCAMBIA3301_NS1DENV2. As in the work of Chaudhury et al. (2007); Khan et al. (2015); Schneider et al. (2015), the floral-dip method was repeated twice to ensure the transformation efficiency and a higher rate of transformants. From seeds obtained in the first generation, 306 were seeded on a plate with a selective medium and 11 germinated. The transformation efficiency obtained in this work was 3.59%, and agrees with the estimation of the original protocol described by Clough and Bent (1998), who determined an efficiency of up to 3%.

The yield obtained in this work, 203 mg of NS1DENV2 per kilogram of fresh leaf, showed that the plant expression system used was efficient and consistent with other systems aimed at optimizing the production of recombinant protein, which obtained, respectively, 70 mg/kg and 24.5 mg/kg (Fedosov et al., 2003; Rigano et al., 2004; Yang et al., 2015; Habibi et al., 2017; Okay and Sezgin, 2018). In their work, Jeong et al. (2018) demonstrated that it is possible to achieve overexpression of the mCherry protein (0.4 mg) per g of *Arabidopsis* callus. Another work using a transient expression method reached a higher yield, however, although transient expression does allow a higher yield, it has the disadvantage of the need for a recurrent transfection step (Garabagi et al., 2012). Some works present higher yields and different molecular strategies to increase the level of expression in plant hosts (Massa et al., 2018). Some highlights are 3.6 g/kg of 2G12 (monoclonal antibody against HIV) in *A. thaliana* (Loos et al., 2011); 4.5 g/kg of GAD65/67 (glutamic acid decarboxylase) in *A. thaliana* seeds (Morandini et al., 2011); 3.7 g/kg GFP in the fresh leaf of *Nicotiana benthamiana* (Yamamoto et al., 2018); among others that were covered in the comparative study of Merlin et al. (2014). In recent work, Diamos et al. (2020) optimized vectors expressing fluorescent proteins and obtained a yield of 3–5 g/kg of fresh leaf in *N. benthamiana*, being ∼50% of the amount of soluble proteins in the leaf. These same vectors were used to the expression of monoclonal antibodies and reached a yield between 1.2 and 1.4 g/kg. Recently, Marques et al. (2020) demonstrated the transient expression of the dengue virus NS1 protein in *N. benthamiana* for diagnostic purposes. Using an elastin-like polypeptide (ELP) as a fusion tag, the authors obtained 445 mg/kg yield, more than double that obtained in this work, but less than other works that used the transient expression approach.

The ELISA results obtained in the present study showed 84.29% sensitivity and 91.43% specificity for anti-dengue IgM, and 83.08% sensitivity and 87.69% specificity for anti-dengue IgG, with high ability to capture anti-dengue antibodies using NS1DENV2 recombinant protein. This result is consistent with other studies already published. The work published by Hunsperger et al. (2014) evaluated commercial diagnostic kits, resulting in anti-dengue IgM in ELISA with specificity of 78–91% and sensitivity 96–98%, including proving that anti-dengue IgM tests are less sensitive in secondary infections. The work of Lima et al. (2012) made the correlation between IgM and IgG reagents with primary dengue infections and subsequent reinfection. They showed that 38% of the primary infections are IgM positive and IgG negative (IgM+ / IgG−).

It is possible to observe a greater dispersion of IgG points in the sample with WT antigens. This occurs because it is a polyclonal primary serum, rich in immunoglobulins with convalescence phase isotypes (IgG), which treat the result as cross reactions which interfere with the specificity of the technique (Fairlie-Clarke et al., 2009). Specificity is the ability to recognize the antibody with its specific antigen, without cross-reactions with non-target molecules. And the expected is that other *Flaviviruses* antigens will not be recognized (Rathore and St John, 2020). However, we still obtained a good result compared to the works cited. Confocal microscopy analysis of *A. thaliana* showed recombinant NS1DENV2 protein localization in foliar tissue labeled with a fluorescent antibody. A primary antibody was used to recognize the polyhistidine tail present in the C-terminus of the recombinant protein. The labeling is evident and positive in the transgenic plant and negative in the wild-type, both receiving the same processing before the microscopic analysis. A difference in development was observed between the transgenic plant and the WT plant (Figure 4B), which suggest that at this point there is a physiological change related to the plant transformation, but that the clarification of this influence would need further investigation (Bent, 2000; Sung and Guy, 2003; Hwang et al., 2017).

Leaves observed from the transgenic clone showed a green fluorescent signal at concentrated points, but spread over the whole leaf. This analysis enabled the authors to hypothesize that the NS1DENV2 protein formed protein storage bodies, which suggest that it looks like a protein factory in the lumen of the RER, and this is in accordance with the results found in works by Stacey et al. (1999); Zang et al. (2009), who also used *A. thaliana* for protein expression and found these protein bodies dispersed in discrete subcellular localizations. In a study by Ishikawa et al. (2015), the expression of transgenic protein in tobacco leaves was shown, which also possessed the retention signal in the RER. Conley et al. (2011) reviewed different strategies for increasing the production of recombinant proteins within cellular subcompartments. Such strategies demonstrate that protein expression within the RER is formed from protein body structures (Joensuu et al., 2010; Gutiérrez et al., 2013; Saberianfar et al., 2016). The results of confocal microscopy demonstrated the formation of fluorescence focal points, consistent with the markings found in this work, reinforcing the previous hypothesis.

In a comprehensive study, Holtz et al. (2015) raised the point that rapid and large-scale manufacture can only be achieved by the technology of the plant-made pharmaceutical (PMP) production platform. Buyel et al. (2017) presented a general approach to scalable process models, in which small-scale models can be used to assess quality indicators quickly and economically, as a step which precedes the full development of a bio-factory in very-large scale. A variety of plants have been evaluated for the ability to produce recombinant proteins on an industrial scale. An ideal expression system must display a high-level of heterologous protein expression, while providing large biomass with an easy and fast growth profile, either in the greenhouse or in the field (Matoba et al., 2011; Xu et al., 2018). *Nicotiana* species present great potential for this purpose. They are widely used in the pre-production stages, in bench studies, and in mass production, both in stable expression systems and in transient systems (Sparrow and Twyman, 2009; Vancanneyt et al., 2009). Sheludko et al. (2007) compared different species of *Nicotiana* (*N. benthamiana, Nicotiana debneyi, Nicotiana excelsior, Nicotiana exigua, Nicotiana maritima, and Nicotiana simulans*) on the production capacity and concluded that *N. excelsior* had the best features in biomass terms.

This study demonstrated that plant antigen production to detect anti-dengue antibodies is a promising alternative to mouse brain and cell culture production of the antigens for diagnostic kits. And it could contribute to the development of future investigations for a rapid test which captures early anti-NS1 antibodies circulating in patients suspected of having this disease. The high yield and low cost for production of the recombinant protein are attractive aspects for commercialization, and thus this technique is a start for the manufacture of diagnostic kits in scalable process models.

## Data Availability Statement

The raw data supporting the conclusions of this article will be made available by the authors, without undue reservation.

## Author Contributions

MX: formal analysis, investigation, methodology, validation, writing – original draft, and writing – review and editing. RD: supervision, visualization, writing – original draft, and writing – review and editing. EF-A and JP: investigation and methodology. CS: resources, supervision, visualization, writing – original draft, and writing – review and editing. SP: project administration, resources, supervision, visualization, writing – original draft, and writing – review and editing. All authors contributed to the article and approved the submitted version.

## Conflict of Interest

The authors declare that the research was conducted in the absence of any commercial or financial relationships that could be construed as a potential conflict of interest.

## Footnotes

1 https://imagej.nih.gov/ij/index.html
