# Supplementary material for: Efficient Plant Production of Recombinant NS1 Protein for Diagnosis of Dengue
Source: Front Plant Sci. 2020 Oct 26;11:581100. doi: 10.3389/fpls.2020.581100 (PMC7649140; doi:10.3389/fpls.2020.581100)
Supplement: Supplementary file 1 [file Data_Sheet_1.docx]

**Supplementary material**

**EFFICIENT PLANT PRODUCTION OF RECOMBINANT NS1 PROTEIN FOR DIAGNOSIS OF DENGUE**

Mariana Fonseca Xisto^1^, Roberto Sousa Dias^1^, Elias Feitosa Araujo^2^, John Willians Oliveira Prates^3^, Cynthia Canedo da Silva^3^, Sérgio Oliveira de Paula^1^

^1^ Department of General Biology, Federal University of Viçosa, Viçosa, Minas Gerais, Brazil.

^2^ Department of Plant Biology, Federal University of Viçosa, Viçosa, Minas Gerais, Brazil.

^3^ Department of Microbiology, Federal University of Viçosa, Viçosa, Minas Gerais, Brazil.

* Corresponding author: e-mail: depaula@ufv.br Av. Peter Henry Rolfs, s / n - University *campus* (UFV), Laboratory of Molecular Immunovirology, Viçosa-MG, 36570-000, Tel: + 55-31-36125015

Key words: Dengue, NS1 protein, *Arabidopsis thaliana,* Diagnosis


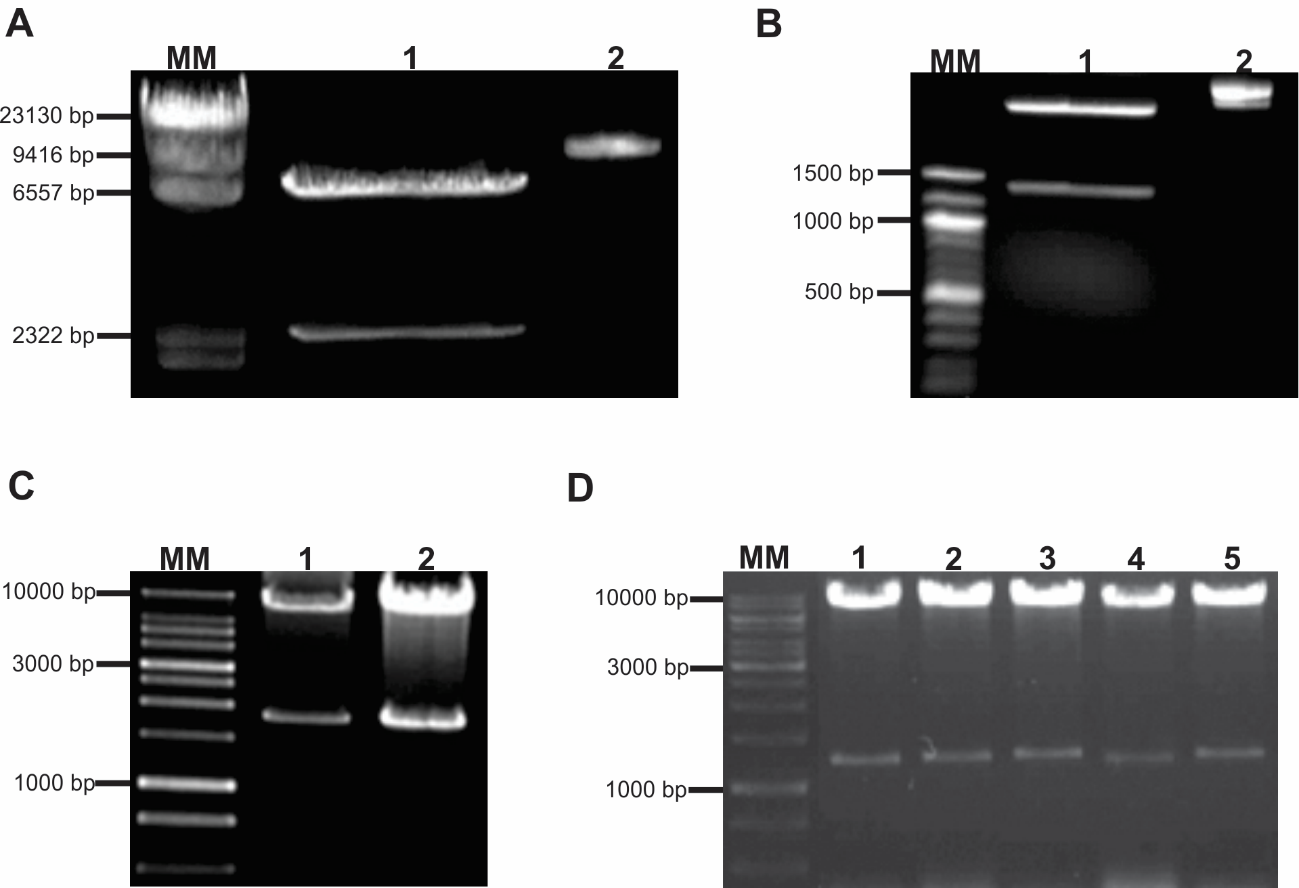


**Supplementary figure 1.** Cloning of the NS1DENV2 gene in Agrobacterium tumefaciens. A) Double vector digestion pCAMBIA3301_empty. MM - Lambda/HindIII DNA molecular marker, 1 - digested plasmid, 2 - undigested plasmid; B) Double vector digest pUC57_NS1DENV2. MM - 100 bp DNA molecular marker, 1 - digested plasmid, 2 - undigested plasmid; C) Confirmation of transformation of E. coli with pCAMBIA3301_NS1DENV2. MM - 1 kb DNA molecular marker, 1-2 - digested plasmids; D) Confirmation of transformation of A. tumefaciens with pCAMBIA3301_NS1DENV2. MM – 1 kb DNA molecular marker, 1-5 - digested plasmids.


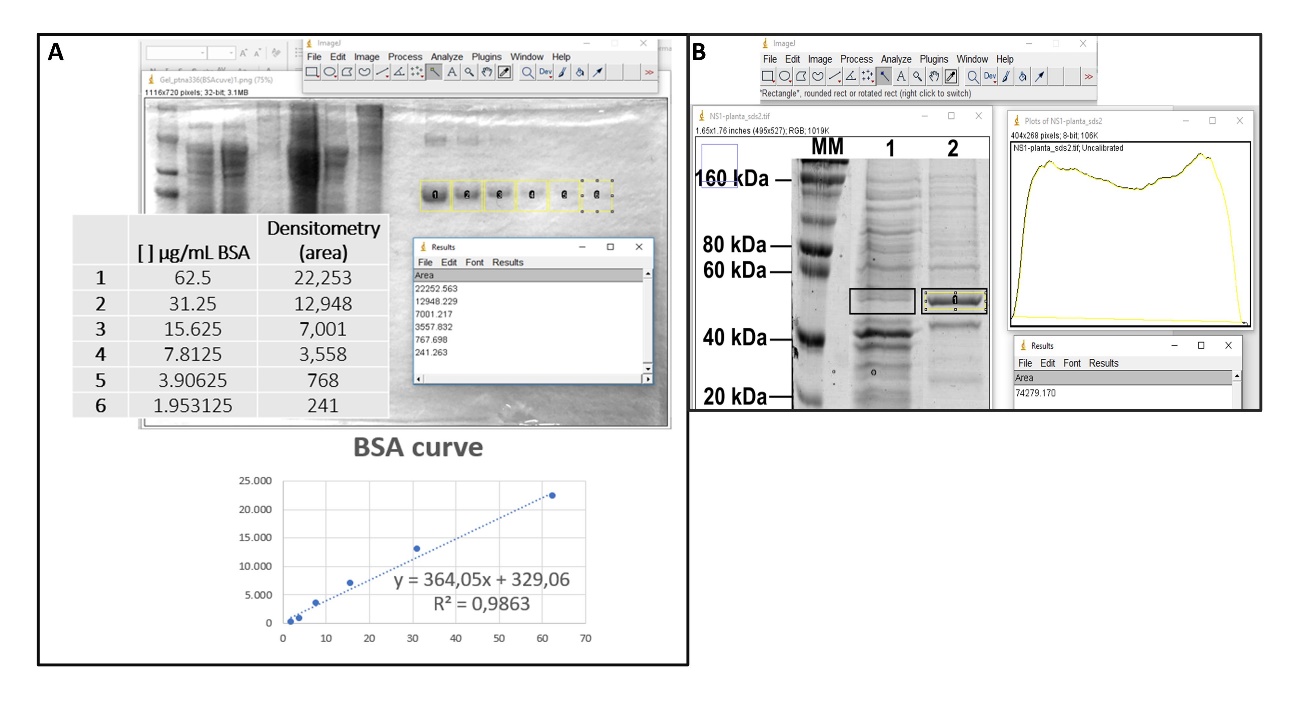


**Supplementary figure 2.** Densitometry analysis. Protein quantification was performed using ImageJ software, based on standard curve. A – Standard curve obtained by bovine serum albumin (BSA) dilution. B – Pixel density of the sample is interpolated with standard curve.

Total yield was obtained by multiplication of the quantity recovery from 1 gr of fresh leaf by the total weight. As resumed by this equation $Y=d x 1000$

Y = yield (mg/kg)

d = protein concentration by densitometry (µg/mL)

as each 1 mL comes from 1 gr of fresh leaf, the protein concentration obtained needs be multiplied by the total grams in one kilogram.
